# Beyond EGFR Targeting in SCCHN: Angiogenesis, PI3K, and Other Molecular Targets

**DOI:** 10.3389/fonc.2019.00074

**Published:** 2019-02-13

**Authors:** Esma Saada-Bouzid, Christophe Le Tourneau

**Affiliations:** ^1^Early Phase Unit, Centre Antoine Lacassagne, Nice, France; ^2^Université Côte d'Azur, Nice, France; ^3^Department of Drug Development & Innovation (D^3^i), Institut Curie, Paris, France; ^4^INSERM U900 Research Unit, Institut Curie, Saint-Cloud, France; ^5^Paris-Saclay University, Saint-Aubin, France

**Keywords:** head and neck cancer, targeted therapy, biomarker, HPV, genomics, clinical trials

## Abstract

Although the molecular landscape of squamous cell carcinoma of the head and neck (SCCHN) has been largely deciphered, only one targeted therapy has been approved to date without any molecular selection, namely cetuximab. Cetuximab is a monoclonal antibody targeting EGFR. It has been shown to improve overall survival in the locally advanced setting in combination with radiotherapy and the recurrent and/or metastatic setting in combination with a platinum compound and 5FU. Beside EGFR targeting agents, antiangiogenic agents have been shown to produce antitumor activity but were associated with substantial toxicity. Buparlisib that targets PI3K was also shown to improve survival in combination with paclitaxel in an unselected patient population. Several other targeted therapies have been developed in SCCHN, most of time in all comers, potentially explaining the limited efficacy reported with them. The recent emergence of clinical trials of targeted therapies in enriched patient populations and precision medicine trials such as umbrella trials might boost the clinical development of targeted therapy in SCCHN.

## KEY CONCEPTS

1. EGFR is the only clinically validated target beside PD-1 in SCCHN.
2. Antiangiogenic agents have been shown to produce antitumor activity in SCCHN but are associated with substantial toxicity.
3. Buparlisib has been the only drug targeting the PI3K/AKT/mTOR pathway to show a survival improvement in SCCHN.
4. There is an urgent need to develop targeted therapies in enriched patient populations in SCCHN.

## Introduction

Tobacco smoking and alcohol consumption are the classical main risk factors of squamous cell carcinoma of the head and neck (SCCHN). The human papilloma virus (HPV) infection has been identified as an additional risk factor for oropharyngeal SCCHN (1). HPV-related SCCHN occur in younger patients, more frequently in men than women, and is associated with a better prognosis. HPV-positive smoking SCCHN patients have an intermediate prognostic (1). Locally advanced SCCHN is treated in a curative intent with a multidisciplinary approach that includes surgery, radiotherapy, and chemotherapy. Despite an improvement in the care of SCCHN patients, almost half of the HPV-negative patients will relapse, most of time within 2 years. Treatment of the relapsing tumors may consist in surgery and/or re-irradiation if possible. Patients with recurrent and/or metastatic (R/M) disease are treated with palliative systemic therapies.

The epidermal growth factor receptor (EGFR) was early on identified as a potential target for the treatment of SCCHN. Indeed, the EGFR protein is almost consistently overexpressed in SCCHN (>90%), and its expression associated with poor prognosis (2, 3). *EGFR* is mutated/amplified in 16% of HPV-negative SCCHN (4). Besides, Cetuximab, a monoclonal targeting the extracellular domain of EGFR, is currently the sole targeted therapy that is approved in combination with a doublet of platinum and 5FU in first-line R/M SCCHN (5). Cetuximab is also approved in combination with radiotherapy for locally advanced SCCHN (6). No predictive biomarker of efficacy of cetuximab has been identified to date in SCCHN, as opposed to colorectal cancer.

We aim to review the main targeted therapies that have been developed beyond cetuximab in R/M SCCHN in light of the molecular landscape of SCCHN.

## Genomic Landscape of SCCHN

The advent of high throughput genomic technologies has enabled to decipher the genomic landscape of SCCHN. SCCHN has a generally high mutational load (7), although this may vary across patients. Several teams reported on the genomic landscape of SCCHN using high throughput technologies (4, 8–11). The Cancer Genome Atlas (TCGA) consortium released the analysis of sequencing data from 279 SCCHN in 2015 (4). The patient population was composed of 243 HPV-negative SCCHN (87%), a majority of men (70%), and mainly heavy smokers. SCCHN of the oral cavity were the most represented tumor location (62%). Following this initial publication, TCGA has reported on more than 500 SCCHN (12).

HPV-positive SCCHN has a rather simple genomic profile (9, 10). HPV-positive SCCHN is characterized by 56% of activating mutations and/or amplifications of the *PIK3CA* gene that encodes for the p100α unit of PI3kinase (PI3K), and a low incidence of tumor suppressor gene (TSG) alterations such as *TP53* mutations (3%) (13), and no *CDKN2A* deletions. HPV-positive SCCHN is also characterized by the dysregulation of transcription factors such as the loss of *TRAF3* (TNF Receptor Associated Factor 3) (22%), and the amplification of *E2F1* (19%). *PIK3CA* mutations were shown to be related to the APOBEC system (apolipoprotein B mRNA editing enzyme, catalytic polypeptide-like) (14), a family of cytosine deaminases that contributes to DNA mutations (12), in HPV-positive SCCHN. APOBEC related mutations were sub-clonal. HPV-negative SCCHN is a more heterogeneous group, with a higher genomic complexity potentially related to tobacco exposure (14). HPV-negative SCCHN is characterized by deleterious mutations and/or homozygous deletions of TSG such as *TP53* (84%) or *CDKN2A* (58%) (4). *PIK3CA* is activated via mutations or gain/amplifications in 34% of cases. Some oncogenes are amplified and include *CCND1* (31%) which encodes for cyclin D1 and controls the G1/S transition of the cell cycle, and *MYC* (14%) which is a transcription factor that regulates the expression of 15% of all genes. Genes coding for tyrosine kinase receptors (TKR) involved in oncogenesis such as *EGFR, FGFR, FGFR, FGFR3, ERBB2, IGF-1R, EPHA2, DDR2*, and *MET* are inconsistently activated (2–15% of cases), most often via amplifications. Conflicting results were reported regarding genomics of HPV-positive smokers. A recent comparison of HPV-positive tumors according to the smoking status found no significant difference in terms of mutation rate and mutation pattern (15), whereas the use of a larger panel showed that HPV-positive oropharyngeal SCC with a smoking history of more than 10 pack-year had a different profile when compared with HPV-positive non-smokers (16). Mutations more frequently associated with smoking status were mutations in *TP53, CDKN2A, KRAS*, and *NOTCH1*. These mutations were associated with poor survival. HLA-A mutations were more common in the non-smokers. These data suggest that smoking history should be taken into account on top of the HPV status, since the biology of HPV-positive HNSCC smoker patients is different than either HPV-positive non-smokers or HPV-negative HNSCC patients.

## Targeting the ErbB Family

EGFR belongs to the ErbB family of tyrosine kinase receptors (TKRs), along with ErbB2 (HER2), ErbB3 (HER3), and ErbB4 (HER4). Binding ligands allow members of the ErbB family to homo- or hetero-dimerize, autophosphorylating the intracellular domain and creating binding sites for signaling proteins. The two primary pathways activated are the RAS/RAF/MEK/ERK and the PI3K/AKT/mTOR pathways. To overcome primary and secondary resistance to EGFR inhibition, a first strategy has been to target other members of the HER (ErbB) family: ErbB2 (HER2), ErbB3 (HER3), and ErbB4 (HER4). *ERBB2*, the second member of the HER family, is amplified in 5% of HPV-negative SCCHN. Lapatinib, a tyrosine kinase inhibitor (TKI) targeting EGFR and HER2, was evaluated in R/M SCCHN (17). Among the 45 enrolled patients, no objective response was observed and 49% of patients experienced adverse events (Aes) (15% grade 3). Afatinib, an irreversible pan-HER TKI, was the first to be evaluated in a phase 3 trial in R/M SCCHN (18). Four hundred eighty-three patients were assigned to afatinib or methotrexate in patients who failed platinum therapy. Median progression-free survival (PFS) was significantly longer in the afatinib arm (2.6 vs. 1.7 months, *p* = 0.03). Because of this modest gain of efficacy and the absence of overall survival (OS) gain, afatinib has not been approved in R/M SCCHN. An increased benefit of afatinib over methotrexate was observed in patients with p16-negative, *EGFR* amplified, HER3-low, and PTEN-high tumors (19), which is being prospectively evaluated in the UPSTREAM umbrella trial (20). Dacomitinib, an oral irreversible pan-HER TKI, was evaluated as first-line treatment in R/M SCCHN (21) in a single-arm phase II trial. Among the 69 enrolled patients, 8 patients achieved a partial response (13%). Median PFS was 3 months, and median OS 7 months. Grade 3/4 diarrhea occurred in 16% of patients leading to frequent dose interruption (41%) and dose reductions (38%). In another phase 2 trial, 10 out of 48 patients (21%) had a partial response (22). Efficacy results were in the same range than the other trial with a median PFS of 3.9 months and a median OS of 6.6 months. The most common AEs were paronychia (65%) and diarrhea (52%). Treatment-related grade 3 AEs occurred in 6 patients. At least one dose interruption and reduction due to treatment-related AEs occurred in 24 patients (50%) and 9 patients (19%), respectively.

Targeting HER3 in SCCHN was also evaluated. Dual-targeting of HER3 and EGFR was evaluated in a randomized phase 2 trial with duligotuzumab, a dual antibody (23). Duligotuzumab was compared to cetuximab in 121 pretreated R/M SCCHN patients. Both drugs were associated with comparable PFS (median: 4.2 vs. 4.0 months), OS (median: 7.2 vs. 8.7 months) and ORR (12 vs. 15%). Patritumab is a fully human anti-HER3 monoclonal antibody. By binding the extracellular domain of HER3, patritumab prevents heregulin-mediated signaling, the dimerization with EGFR or HER2, and promotes the receptor internalization and degradation. A randomized phase 2 study evaluated the combination of cetuximab and platinum chemotherapy with patritumab or placebo in first-line R/M SCCHN (24). AEs were more frequent with patritumab than placebo, leading to discontinuation in 16% of patients treated with patritumab vs. 5% with placebo. The addition of patritumab was not associated with a gain of efficacy in terms of overall response rate (ORR) (36 vs. 28%) or PFS (5.6 vs. 5.5 months).

In summary, cetuximab has been the first and unique approved therapy targeting the ErbB pathway. No validated biomarker has been identified. Targeting other members of the ErbB family is associated with disappointing efficacy probably due to the lack of molecular selection.

## Targeting Angiogenesis

The vascular endothelial growth factor (VEGF) and its receptors VEGFR2 and VEGFR3 are overexpressed in SCCHN (25, 26). VEGF overexpression is associated with poor survival (27). TKIs targeting VEGFR demonstrated limited activity in pre-treated R/M SCCHN with ORR never exceeding 10% (28–30) (Table 1). In addition, serious safety issues were frequently reported, including grade 3–4 fatigue in 20–30% of patients, hand foot syndrome and diarrhea with sorafenib (28), and severe bleeding events with sunitinib (4 deaths out of 38 patients) (29). Only 19 out of 30 patients treated with axitinib received the full planned dose (30). Antiangiogenic agents were also combined with other targeted therapies and/or cytotoxic agents (Table 1). Although the results of some single arm phase 2 trials with sorafenib as single agent were encouraging (33, 34), the addition of sorafenib to cetuximab did not improve the ORR in randomized phase 2 trials as compared to cetuximab alone (31). The addition of vandetanib to docetaxel did even worse in terms of PFS as compared to docetaxel alone (32). A phase 3 trial assessed the efficacy of the addition of bevacizumab that is a monoclonal antibody targeting VEGF to a doublet of platinum with 5FU or docetaxel. The trial did not reach its primary endpoint with a median OS of 11 months in the control arm vs. 12.6 months in the experimental arm, but showed an improved PFS and ORR with the addition of bevacizumab. Grade 3–5 AEs were more frequent with bevacizumab (67 vs. 82%, *p* = 0.0003), especially for grade 5 bleeding (0 vs. 2.6%, *p* = 0.03). The limited efficacy with substantial toxicity has clearly impacted the clinical development of antiangiogenic agents in SCCHN patients, although combinations of antiangiogenic agents with immunotherapy are ongoing (36).

**Table 1:** Selected clinical trials evaluating antiangiogenic agents in SCCHN patients.

	**Phase**	**N**	**ORR (%)**	**Median PFS (mo)**	**Median OS (mo)**
Sorafenib (28)	II	23	5	3.4	8
Sunitinib (29)	II	38	3	2	3.3
Axitinib (30)	II	42	7	3.7	11
Cetuximab + sorafenib	II	27	8	3	9
Cetuximab (31)		28	8	3.2	5.7
Docetaxel + vandetanib	II	15	13	0.7	6.8
Docetaxel (32)		14	7	2.3	6
Cetuximab + bevacizumab (33)	II	46	16	2.8	7.5
Pemetrexed + Bevacizumab (34)	II	40	30	5	11.3
Platinum + 5FU or docetaxel	III	200	25	4.4	11.0
Platinum + 5FU or docetaxel + bevacizumab (35)		203	36	6.1	12.6

*ORR, overall response rate; PFS, progression-free survival; OS, overall survival; mo, months*.

## Targeting the PI3K/AKT/mTOR Pathway

Alterations in the PI3K/AKT/mTOR pathway are among the most frequent in SCCHN (13% to 56%), regardless of the HPV status (4). *PIK3CA* amplifications were reported in pre-malignant and cancer lesions, suggesting an early role in SCCHN carcinogenesis (37). The activating mutations of *PIK3CA* are reported in 6–8% of HNSSC, 73% of these mutations being localized in 3 hotspots, namely E542K and E545K coding for the helical domain, and H1047R/L in the kinase domain (38). These three mutations are associated with the overexpression of the protein (39). The function of the other mutations, which are not uncommon, is more uncertain (7, 40). Many other deregulations have been reported in the PI3K/AKT/mTOR pathway including alterations of PTEN, PIK3R1, and mTOR. Preclinical data showed that patient-derived tumorgrafts with *PIK3CA* mutations were sensitive to PI3K targeting, as opposed to *PIK3CA*-wild-type tumorgrafts (38). Wirtz et al. reported that engineered cell lines harboring the hotspot E545K and H1047R *PIK3CA* mutations were less sensitive to PI3K inhibition (41). In contrast, another study found that the H1047R-expressing cell lines had increased sensitivity to PI3K inhibition, whereas those expressing E545K showed slightly increased sensitivity. These conflicting results open the debate on the actual oncogenic addiction of *PIK3CA* mutations, their actual weight when compared with other driver mutations, and highlight the difficulty of targeting this pathway.

The first results of clinical trials targeting the PI3K/AKT/mTOR pathway with non-selective inhibitors were disappointing (Table 2). mTOR inhibitors were first evaluated. In phase 2 trials, no responses were observed with everolimus (42) or temsirolimus (43). The combination of erlotinib with everolimus (44) or temsirolimus (45) resulted in increased toxicity without any additional efficacy. PX-866, an oral, irreversible, pan-isoform inhibitor of PI3K, was evaluated in combination with docetaxel in a phase 2 randomized trial (46). When compared with docetaxel alone, the combination of PX-866 with docetaxel did not improve the PFS, ORR, and OS. PX-866 was also evaluated in combination with cetuximab in another randomized phase 2 trial in pretreated R/M SCCHN (47). The combination again did not improved ORR, PFS, and OS. Buparlisib, a selective PI3K inhibitor of p110α/β/δ/γ subunit was first tested as a single agent in pretreated R/M SCCHN (49). Preliminary results showed a 39% disease control rate at 2 months in patients whose tumor did not have *PIK3CA* mutation (49). Buparlisib was further evaluated in 2nd line R/M SCCHN in combination with weekly paclitaxel in BERIL-1, a randomized placebo controlled phase 2 trial (48). The median PFS was significantly longer in the buparlisib arm (4.6 vs. 3.5 months, *p* = 0.01), as well as OS (10.4 vs. 6.5 months, *p* = 0.041). However, grade 3–4 Aes were more frequent with buparlisib, especially in terms of hyperglycemia (22%), anemia (18%), neutropenia (17%), and stomatitis (9%). Beril-1 was the first randomized trial to demonstrate a significant improvement in PFS and OS with a PI3K inhibitor in R/M SCCHN, but at the price of high toxicity. A preplanned exploratory analysis showed that the combination seemed to benefit a subgroup of patients with *TP53* alterations, HPV-negative status, low mutational load, or high infiltration of tumor infiltrating lymphocytes (TILs) or CD8-positive cells) (50). Importantly, the outcome of patients treated with buparlisib was not associated with deregulation of the PI3K/AKT/mTOR pathway (*PIK3CA* mutation/amplification, PTEN loss). A potential mechanism of action of buparlisib is the promotion of the anti-tumor immune response through the promotion of the INFγ secretion (50). A phase 3 trial is currently planned and will evaluate the predictive value of these biomarkers. To further improve the efficacy of targeting the PI3K pathway, selective PI3K inhibitors are currently developed (51).

**Table 2:** Selected clinical trials evaluating inhibitors of the PI3K/AKT/mTOR pathway in SCCHN patients.

	**Phase**	***N***	**ORR (%)**	**Median PFS (mo)**	**Median OS (mo)**
**mTOR INHIBITORS**					
Everolimus (42)	II	9	0	1.5	4.5
Temsirolimus (43)	II	40	0	2	3.7
Erlotinib + everolimus (44)	II	35	2.8	3	10.2
Erlotinib + temsirolimus (45)	II	12	0	1.9	4
**PI3K INHIBITORS**					
Docetaxel	II	43	5	2.7	6.5
Docetaxel + PX-866 (46)		42	14	3.1	8.8
Cetuximab	II	41	7	2.7	8.5
Cetuximab + PX-866 (47)		42	4	2.7	7.0
Paclitaxel + placebo	II	79	14	3.5	6.5
Paclitaxel+ buparlisib (48)		79	39	4.6^*^	10.4^*^

* *p < 0.05*.

## Other Molecular Targets

### Targeting RAS

The proportion of SCCHN having a *KRAS* mutation is low around 5% (52, 53). The activating mutations of *HRAS*, similarly rare in the population of Caucasian HPV-negative SCCHN (5%) (4, 8, 11, 38), are more frequent in the oral cavity SCC of Asian populations because of chewed betel nut (9, 54, 55), and snuff (56). The RAS proteins must undergo a series of post-translational modifications, and in particular a farnesylation, to be functional. Inhibition of farnesyl transferase activity produced antitumor activity in preclinical models of SCC of the skin with *HRAS* mutations, an antitumor effect that was not observed in models with *NRAS* or *KRAS* mutations (57). Tipifarnib, a farnesyl transferase inhibitor is currently tested in a phase 2 study in advanced tumors with activating mutations of *HRAS* (58). Preliminary reports of 7 evaluable SCCHN showed 5 patients (71%) achieving a partial response with a median duration of response of 14.1 months. No *HRAS* mutated SCCHN patients experienced an objective response on their last therapy prior to receiving tipifarnib. If these results are confirmed in the ongoing phase II KO-TIP 007 trial (NCT03719690), tipifarnib could become a standard in this rare and aggressive subgroup of patients.

### Targeting the Cell Cycle Regulators

The majority of HPV-negative SCCHN harbors genetic alterations involving the cell cycle such as *TP53* mutations, *CCND1* amplification, *CDKN2A* deletion, and p16 inactivation. These later deregulations enable to circumvent the mitotic checkpoints through aberrant cyclin-dependent kinase activation. *CCND1* is amplified in 31% of HPV-negative SCCHN and is involved in the cell cycle with CDK4/CDK6 in G1 phase, and in G1/S transition (59). Several clinical trials evaluate CDK4/CDK6 inhibitors as monotherapy [palbociclib (NCT03088059), ribociclib (NCT03179956), abemaciclib (NCT03356587)] or in combination with other targeted therapies, such as the combination of palbociclib with cetuximab (NCT02499120) or the combination of palbociclib with gedatolisib that is a dual PI3K/mTOR inhibitor (NCT03065062). The inclusion in these trials is usually restricted to HPV-negative HNSCC and sometimes to patients whose tumors harbor alterations in the genes involved in cell cycle regulation (amplification of *CCND1* in NCT03088059, intact Rb and genetic alterations in CDK4/CDK6 pathway in NCT03356587). The combination of palbociclib with weekly cetuximab was shown to be safe in a phase I trial (60) with no dose-limiting toxicity. The phase II trial enrolled 30 pretreated HPV-negative R/M SCCHN patients. Among the 28 evaluable reported patients, 3 patients had a complete response (11%), and 8 patients a partial response (29%).The median PFS was 5.4 months and the median OS 9.5 months (61). A randomized phase II trial evaluating this combination (PALATINUS, NCT02499120) is ongoing. Despite encouraging results with CDK4/6 inhibitors in R/M SCCHN, the oral route of these molecules constitutes a significant limit for a development in the treatment of R/M SCCHN, since many patients are no longer able to swallow.

### Targeting IGF1 Receptor

IGF-1R is mutated or amplified in 4% of HPV-negative SCCHN (4). Although preclinical data supported IGF-1R inhibition in SCCHN cell lines (62), figitumumab or cixutumumab, monoclonal antibodies targeting IGF-1R, had no efficacy in unselected patient populations (63, 64).

### Targeting FGF Receptors

The *FGFR 1, 2*, and *3* (Fibroblast Growth Factor Receptor) activating mutations/amplifications were reported in 14% of the HPV-negative SCCHN (4)*. FGFR3* fusion genes have also been reported in HPV-positive SCCHN (4). Specifically, the *FGFR3-TACC3* fusion gene has been evaluated in preclinical models (65). Exposure of carcinoma models carrying *FGFR3* fusion genes after exposure to the FGFR inhibitor PD173074 resulted in significant antitumor activity, an effect that was not observed in cell lines with *FGFR3* activating mutation. Several trials are testing FGFR inhibitors in molecularly selected patients (NCT02706691; NCT03088059).

### Targeting MET

The *MET* (Mesenchymal Epithelial Transition) gene encodes a TKR which is activated by binding to its ligand, the Hepatocyte Growth Factor (HGF). The common overexpression of MET in SCCHN is associated with a poor prognosis and resistance to cetuximab (66). Despite a strong rationale to counteract resistance to EGFR inhibitor by targeting MET,results were disappointing in unselected populations. A phase II trial evaluating foretinib (67), a TKI targeting MET, stopped at first interim analysis because no objective response was observed in the first 14 unselected R/M SCCHN patients. A randomized phase II trial that compared the efficacy of tovantinib, another TKI targeting MET, in combination with cetuximab to cetuximab alone failed to show any significant difference in terms of ORR, PFS, or OS (68).

### Targeting MYC

The *MYC* gene produces a transcription factor that regulates the expression of 15% of genes by binding to Enhancer Box sequences (E-boxes), and by recruiting enzymes capable of acetylating lysine amino acids from histones such as histone acetyltransferases. *MYC* is amplified in 14% of HPV-negative SCCHN (4). Bromodomain and terminal domain (BET) inhibitors are currently evaluated in cancer patients with *MYC* amplifications (69) (NCT02419417).

### Targeting Tumor Suppressor Genes

Oncogene abnormalities that can be targeted with currently available drugs are present in a minority of SCCHN patients. In contrast, the vast majority of SCCHN have a loss of function of tumor suppressor genes (TSGs) such as *TP53* and *CDKN2A*. In HPV-negative SCCHN, this loss is due to inactivating mutations and/or deletions of the genes themselves. In HPV-positive SCCHN, the E6 viral oncoprotein prevents the induction of apoptosis by indirect p53 degradation. The targeting of TSGs is less intuitive than that of oncogenes (70). Unlike oncogene mutations, those of TSGs have to be recessive to result in a loss of function of the protein. Many studies carried out in oncology show that the loss of heterozygosity may be sufficient, by a phenomenon of dosage, to contribute to the cellular transformation. The integration of this information is key since the data from the pan-tumoral sequencing analysis revealed that the majority of chromosome region copy number variations were deletions and that the majority of genes involved were TSGs (71).

Sixty to 100% of HPV-negative SCCHNs have inactivating *TP53* mutations (9–12). These mutations are distributed quite homogeneously along the gene with some hotspots. Several approaches have been developed to target TP53 loss of function (72–74). APR-246 is a small molecule capable of restoring the conformation of mutated p53 proteins in wild conformation (75, 76). In SCCHN, the effect of APR-246 was evaluated in 4 different SCCHN cell lines (77). Reactivation of p53 was observed in PRIMA-1 or CP-31398 treated cell lines, and in wild *TP53*-treated cell lines treated with nutlin-3. Used in combination, these small molecules increased the cytotoxicity of cisplatin, 5-fluorouracil, paclitaxel, and erlotinib (77).

## Conclusions

The characterization of the molecular landscape of SCCHN allowed the identification of actionable and potentially targetable genomic alterations. Despite this undeniable advance, very few targeted therapies have shown a significant efficacy in unselected R/M SCCHN. One potential explanation for this is the lack of clinical trials performed in molecularly enriched patient populations. The UPSTREAM trial is an umbrella biomarker-driven study dedicated to R/M SCCHN patients, sponsored by the European Organization of Research and Treatment of Cancer (20). The UPSTREAM is the first precision medicine trial in SCCHN. In this trial, patients have to undergo a mandatory fresh biopsy in order to establish the molecular profile of patients' tumors. Patients are then allocated to either a targeted therapy or immunotherapy cohort in the absence of those biomarkers. Tissue of origin agnostic trials, such as NCI-MATCH (NCT02465060) or TAPUR (NCT02693535), are also interesting ways to evaluate targeted therapies in enriched HNSCC patients.

## Author Contributions

All authors listed have made a substantial, direct and intellectual contribution to the work, and approved it for publication.

### Conflict of Interest Statement

CL has participated in advisory boards of BMS, MSD, Merck Serono, Amgen, Novartis, Nanobiotix, Astra Zeneca. ES-B has participated in advisory boards of BMS, Merck Serono, Astra Zeneca.

## Abbreviations

Aes: adverse events
EGFR: epidermal growth factor receptor
HPV: Human papilloma virus
ORR: overall response rate
OS: overall survival
PFS: progression free survival
R/M: recurrent metastatic
SCCHN: squamous cell carcinoma of the head and neck
TKR: tyrosine kinase receptor
TKI: tyrosine kinase inhibitor
TSG: tumor suppressor gene.
